# Epidemiological trend of chikungunya outbreak in Pakistan: 2016–2018

**DOI:** 10.1371/journal.pntd.0007118

**Published:** 2019-04-18

**Authors:** Nazish Badar, Muhammad Salman, Jamil Ansari, Aamer Ikram, Javaria Qazi, Muhammad Masroor Alam

**Affiliations:** 1 Department of Virology, National Institute of Health, Chak Shahzad, Islamabad, Pakistan; 2 Department of Biotechnology, Quaid-i-Azam University, Islamabad, Pakistan; Naval Medical Research Center, UNITED STATES

An unprecedented expansion of vector-borne diseases, especially those caused by viruses transmitted through mosquitoes, has posed a serious public health challenge worldwide. Chikungunya virus (CHIKV), which mainly invades tropical and subtropical regions, is one of the recently emerging pathogens associated with severe morbidity in humans. Although Pakistan is known to be endemic for arboviral infections, only Crimean-Congo hemorrhagic fever (CCHF) and dengue have been officially recognized over the last three decades, despite a recent trend of increasingly frequent chikungunya cases, first detected in December 2016.

**Fig 1:**
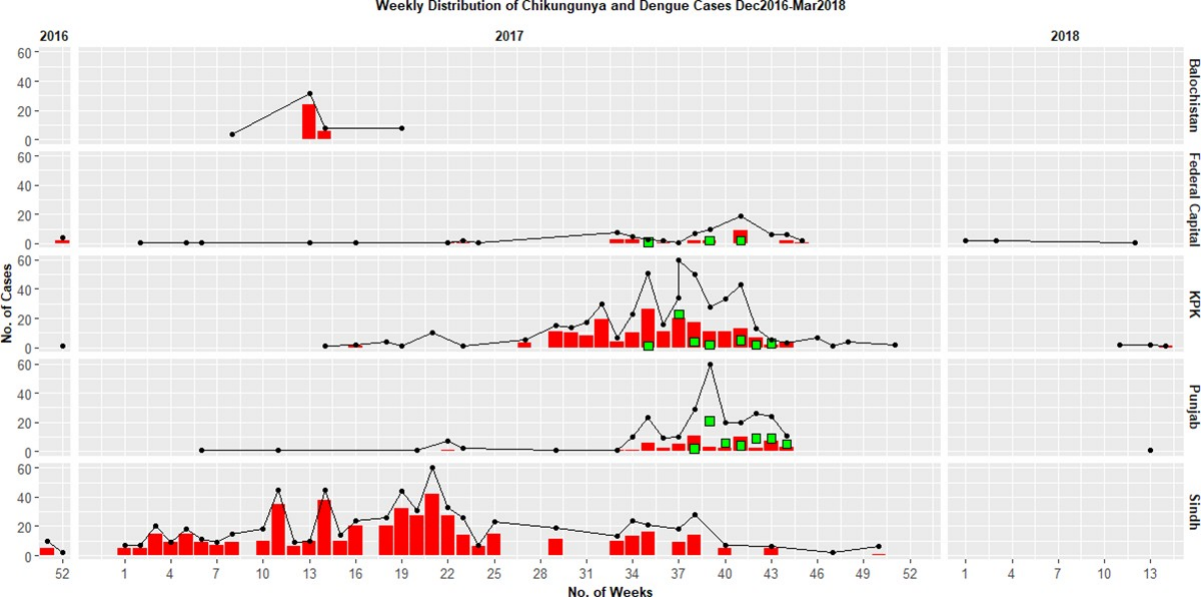
Epidemiological curve of chikungunya cases reported at NIH, Islamabad. Red bars indicate the number of patients who tested positive for chikungunya RNA using real-time reverse transcriptase PCR, green squares indicate count of dengue-positive patients, and black lines represent the total number of reported cases at NIH. x-Axis shows the number of weeks, starting from week 51 in 2016 to week 14 in 2018. The mean age of chikungunya-positive patients was 31.8 ± 15.7 years, with a male-to-female ratio of 1:1.2 (*P* value = 0.53). KPK, Khyber Pakhtunkhwa; NIH, National Institute of Health.

Chikungunya infection is not new to Pakistan, as the presence of anti-CHIKV antibodies has been reported in rodent and human sera since the 1980s [1]. After a lapse of three decades, the first cases were reported from Sindh Province and the Federal Capital, with cases expanding to three provinces by mid-2017 (Fig 1). A sharp decline in cases was observed by the end of 2017, after the onset of winter, leading to restricted vector activity. We hereby present an update on CHIKV testing at National Institute of Health in Islamabad, the only public-sector laboratory at the federal level providing free diagnostic services for pathogenic arboviruses. Interestingly, utilization of the Trioplex real-time PCR [2] assay helped us to screen samples for two additional flaviviruses (dengue and Zika virus), making this the first ever study on Zika virus screening from Pakistan. Out of a total 1,549 samples tested through July 31, 2018, we found chikungunya and dengue infection in 776 (50%) and 109 (7%) patients, respectively, without a single patient positive for Zika virus. Our data further reflect the importance of population mobility and the abundance of potential mosquito vectors (*Aedes aegypti* and *A*. *albopictus*) as key factors favoring efficient virus transmission in Pakistan. Likewise, even though this is the first CHIKV outbreak reported at a vast geographical scale in Pakistan, identification of concomitant cases across all four provinces indicates an increased gravity of the burden of mosquito-borne diseases in the coming years.

Without implementation of a nationwide functional surveillance system, thorough investigations of disease determinants cannot be achieved to preempt disease trends and initiate control measures before they affect large populations. A sustainable surveillance program with potential epidemic indicators is also required to reduce the risk of virus transmission by generating early warnings, which is among the best-known interventions considering the fact that impeccable vector control is not feasible. Although Pakistan is known to be at high risk for arboviral infections, with unswerving epidemics of dengue and CCHF each year, the disease prevention and control measures are still below par. The very recent CHIKV outbreak, initially perceived as a “mysterious” pathogen [3], is a testament to debilitated preparedness plans existing at the local and regional scale. Considering the fact that humans are the only amplifying host for such anthroponotic pathogens, the most effective interventions, such as vector control and isolation and restriction of patients to terminate further transmission, may not be readily achievable in resource-poor countries like Pakistan; thus, it is imperative to identify areas with the greatest risk of infection to inform and implement control prioritization measures. Our findings further highlight the strengthening of laboratory services at provincial and at-risk districts to generate accurate information for patient management and sensible allocation of health resources.
